# On the weekly cycle of atmospheric ammonia over European agricultural hotspots

**DOI:** 10.1038/s41598-022-15836-w

**Published:** 2022-07-19

**Authors:** Martin Van Damme, Lieven Clarisse, Trissevgeni Stavrakou, Roy Wichink Kruit, Louise Sellekaerts, Camille Viatte, Cathy Clerbaux, Pierre-François Coheur

**Affiliations:** 1 Université libre de Bruxelles (ULB), Spectroscopy, Quantum Chemistry and Atmospheric Remote Sensing (SQUARES), Brussels, Belgium; 2 Royal Belgian Institute for Space Aeronomy, Brussels, Belgium; 3 National Institute for Public Health and the Environment (RIVM), Bilthoven, The Netherlands; 4 LATMOS/IPSL, Sorbonne Université, UVSQ, CNRS, Paris, France

**Keywords:** Atmospheric chemistry, Environmental monitoring, Atmospheric science, Element cycles

## Abstract

The presence of a weekly cycle in the abundance of an atmospheric constituent is a typical fingerprint for the anthropogenic nature of its emission sources. However, while ammonia is mainly emitted as a consequence of human activities, a weekly cycle has never been detected in its abundances at large scale. We expose here for the first time the presence of a weekend effect in the NH_3_ total columns measured by the IASI satellite sounder over the main agricultural source regions in Europe: northwestern Europe (Belgium-the Netherlands-northwest Germany), the Po Valley, Brittany, and, to a lesser extent, the Ebro Valley. A decrease of 15% relative to the weekly mean is seen on Sunday–Monday observations in northwestern Europe, as a result of reduced NH_3_ emissions over the weekend. This is confirmed by in situ NH_3_ concentration data from the National Air Quality Monitoring Network in the Netherlands, where an average reduction of 10% is found around midnight on Sunday. The identified weekend effect presents a strong seasonal variability, with two peaks, one in spring and one in summer, coinciding with the two main (manure) fertilization periods. In spring, a reduction on Sunday–Monday up to 53 and 26% is found in the NH_3_ satellite columns and in situ concentrations, respectively, as fertilization largely drives atmospheric NH_3_ abundances at this time of the year.

## Introduction

Weekly recurring patterns in measured data can usually be traced back to human activities, as natural processes do not occur at this temporal scale^1^. Strong weekly cycles are expected in parameters related to air quality, due to differences in intensity of emission sources between week and weekend days related to work legislation and/or religious practices. A “Sunday effect” was first observed in photochemical air pollution in the seventies, resulting from reduced industrial activities and traffic during this rest day in the United States^2^. More recently, the availability of satellite measurements allowed the detection of weekly cycles and the influence of anthropogenic activities on the environment at regional and global scales. As an example, using MODIS data, religious affiliation has been identified as a driver of the weekly cycle detected in the fire activity in sub-saharian Africa^3^.

Perhaps the best documented weekly cycle is the one of nitrogen dioxide (NO$_2$). Ground-based, aircraft and satellite measurements have shown that NO$_2$ abundances exhibit a strong weekly cycle in industrialized regions and large cities^4–6^. This is expected, as the main NO$_x$ emission source is fossil fuel combustion related to traffic and industrial activities. In Europe, a reduction of 25–50% has been reported on Sunday over several cities based on GOME satellite NO$_2$ column observations (1996–2001)^7^. The amplitude of this reduction even reached 60% in Milan, Italy. A weakening of the NO$_2$ weekly cycle magnitude was identified in the long term time-series (2005–2017) offered by the OMI instrument, over regions presenting a reduction in anthropogenic emissions, implying an increased importance of background emissions^8^.

Here we investigate for the first time the daily variability over Europe of ammonia (NH_3_), another important nitrogen species. As agricultural sources (from stables, feedlots, fields) dominate its emission fluxes, the presence of a significant weekly cycle is not necessarily expected. A few ground-based studies however have already identified a reduction of NH_3_ abundances during weekends in large cities where traffic is a significant contributor to the emissions (e.g.,^9–12^). By contrast, Wang et al.^13^ report the absence of an NH_3_ weekly cycle at an urban site near Shanghai, while nearby rural and industrial sites exhibit a reduction of in situ concentrations on Saturday and Sunday. To assess the potential presence of weekly cycles in NH_3_ abundances over Europe, we have used here satellite and ground-based observations.

## Dataset and statistical method

### IASI satellite measurements

For over a decade, satellite instruments operating in the infrared provide daily distributions of NH_3_ at the global scale^14–17^. This work is based on 13 years (2008–2020) of observations from the Infrared Atmospheric Sounding Interferometer (IASI) mission, which is composed of three identical instruments on-board the Metop-A, -B, -C platforms launched in 2006, 2012 and 2018, respectively. Each instrument samples the Earth globally two times per day, with a footprint of 12 km at nadir and a morning overpass time at 9:30 am when crossing the equator and an evening one at 9:30 pm. In total we use twelve years (2008–2019) of IASI-A, eight years (2013–2020) of IASI-B and one year (2020) of IASI-C from the Artificial Neural Network for IASI (ANNI) NH$_{3}$ version 3.2 reanalyzed dataset (see^18–21^ for more details on the ANNI-NH_3_ retrieval, the near-real time and reanalyzed datasets and validation work). Only the morning overpasses have been considered as infrared measurements are more sensitive to the lowest layers of the atmosphere at this time of the day^22^.

**Figure 1:**
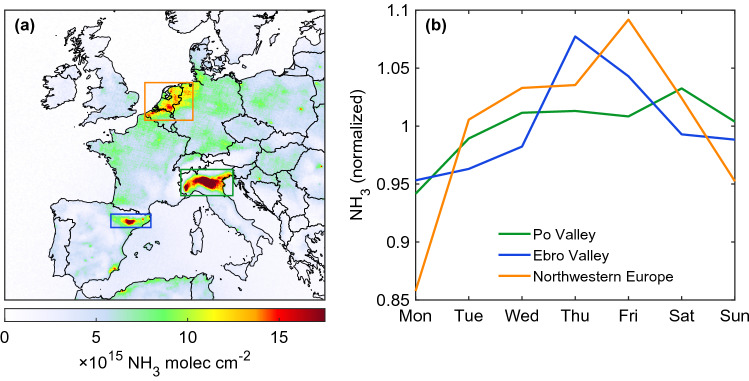
(**a**) Average NH_3_ total column distribution (molec cm$^{-2}$) over Europe based on IASI-A (2008–2019), IASI-B (2013–2020) and IASI-C (2020) morning observations. (**b**) Normalized NH_3_ day of the week time-series over the three main source regions in Europe (indicated by coloured rectangles in panel (**a**)); only continental data are considered.

The average NH_3_ distribution over Europe for the period of interest is shown in Fig. 1a and highlights the Po Valley (Italy), the Ebro Valley (Spain) and the northwestern Europe (including north of Belgium, the Netherlands and northwestern Germany) as main source regions (coloured rectangles in Fig. 1a). Localised maxima over industrial point sources are also seen over Pulawy (Poland), Targu Mures (Romania) and Kutina (Croatia)^23,24^. In Europe, 94% of the reported NH_3_ emissions are from agricultural sources^25^. In Lombardy, where the highest NH_3_ columns are reported by IASI over the Po Valley, about 90% of the NH_3_ emissions originate from manure management^26^. The Ebro Valley is characterized by intensive agricultural activities^27,28^ and the Aragon and Catalonia regions together account for more than half of the pig herd in the country^29^. Finally, northwestern Europe is a well-known region of intensive agriculture, characterized by the highest dairy cow, beef cattle, pig and chicken densities in Europe^30,31^.

### LML ground-based network

The Netherlands is one of the first countries with a national monitoring network for NH_3_ enabling the assessment of the efficiency of implemented emission abatement policies^32,33^. The hourly temporal sampling of the NH_3_ measurements performed by the National Air Quality Monitoring Network (or LML standing for “Landelijk Meetnet Luchtkwaliteit”) allows us to investigate the weekly cycle of NH_3_ from the ground perspective. Observations from 2008 to 2018 have been considered from the eight LML stations shown in Fig. 3a. These are located in high (Vredepeel-131 (2008–2018), Wekerom-738 (2008–2018)), moderate (Valthermond-929 (2008–2017), Zegveld-633 (2008–2018), Eibergen-722 (2008–2014)) and low emission regions (De Zilk-444 (2008–2018), Huijbergen-235 (2008–2014), Wieringerwerf-538) (2008–2018))^33^. Additional information on the monitoring sites and the instrumentation used can be found in Berkhout et al.^34^.

### Mann-Whitney test

Daniel et al.^35^ detail the importance of an appropriate statistical analysis to avoid erroneous conclusions on the presence or absence of a weekly cycle due to human influence. The common procedure, widely applied to the identification of weekly cycles in meteorological variables, is to test the null hypothesis and reject it based on a threshold on the significance level^36^. Two-tailed t-test, which require a normally distributed dataset, have been used to confirm^37^ or infirm^38^ the detection of a weekly cycle. In this work, the Mann-Whitney test, or Wilcoxon rank sum test, is used. It is a nonparametric test to evaluate whether two independent samples come from the same distributions with equal medians, and returns the associated *p*-value. The *p*-value expresses the probability to encounter the null hypothesis, when the medians of the two samples are equal. Here, we consider a weekly cycle to be significant if the *p*-value is lower than 0.01.

## Results

### The IASI satellite view of the NH_3_ weekly cycle

To investigate the possible presence of a weekly cycle, we first look at the three main NH_3_ hotspots in Europe. The NH_3_ weekly cycles based on 2008–2020 IASI morning data over land are shown in Fig. 1b and represents a first identification of a weekly cycle over the Po Valley, the Ebro Valley and the northwestern Europe. Here we normalize the time-series by dividing by the mean of all data. The weekly cycles present minima on Monday for the three hotspots. The Ebro Valley and the northwestern Europe are characterized by a similar intra-weekly variability, with NH_3_ column maxima on Thursday-Friday and values starting to decrease on Saturday. The Po Valley presents a distinct maximum on Saturday and the decrease in NH_3_ abundances only starts on Sunday. The weekly cycle in NH_3_ total columns over northwestern Europe is characterized by the largest amplitude, with Monday observations being 14% lower than the observations over the entire week. The weekend effect therefore consists here in the NH_3_ abundances starting to decrease on Saturday and decreasing further on Sunday and Monday. While the fact that the minimum is reached on Sunday–Monday instead of Saturday–Sunday could be interpreted as the result of the morning overpass time of the IASI satellite, this hypothesis will be rejected in the next section.

**Figure 2:**
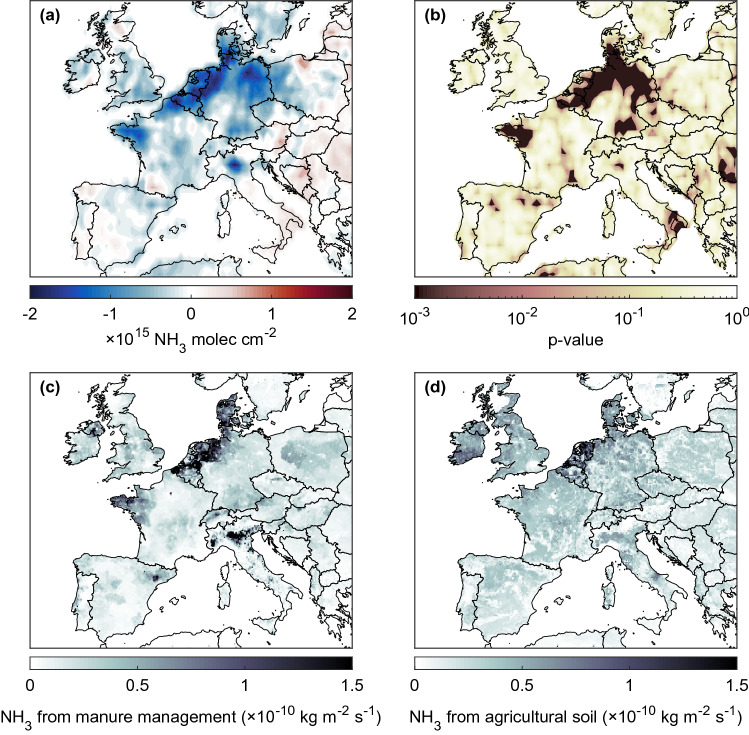
(**a**) Distribution over Europe of the difference in NH_3_ columns (molec cm$^{-2}$) between the average of the Sunday–Monday IASI morning observations (2008–2020) and the average for the rest of the week (weekend effect). (**b**) Distribution of the associated *p*-value calculated with the Mann-Whitney test to assess whether the magnitude of the weekend effect observed is significant (0.5$^{\circ }$ $\times$ 0.5$^{\circ }$ grid). (**c**–**d**) NH_3_ emissions (kg m$^{-2}$ s$^{-1}$) from manure management and agricultural soil in 2015 from the Emissions Database for Global Atmospheric Research (EDGAR)^39^(0.1$^{\circ }$ $\times$ 0.1$^{\circ }$ grid).

Given these first results, we focus in Fig. 2 on the distribution of the identified weekend effect, defined as the average difference in NH_3_ total column over Europe between the Sunday–Monday observations and the rest of the week^35^. A strong weekly cycle is unambiguously identified in several parts of Europe. In the Netherlands, a drop close to 2 $\times$ 10$^{15}$ molec cm$^{-2}$ is seen for these days, which represents an average decrease of 15% over the northwestern Europe (50$^{\circ }$N–55$^{\circ }$N; 2$^{\circ }$E–15$^{\circ }$E). In addition to the latter and the Po Valley, Brittany (France) is also characterized by a strong decrease in NH_3_ total columns on Sunday–Monday. To assess the significance of the observed weekly cycle, the Mann-Whitney test has been applied to the data included in each cell of this distribution, considering the Sunday–Monday observations as one set of data and the Tuesday to Saturday observations as the other set. The resulting distribution of the *p*-values (Fig. 2b) confirms the presence of a marked (*p* < 0.001) weekend effect in the northwestern Europe, Brittany and to lesser extent in the Ebro and Po Valleys. It also shows that the moderate weekly cycle reported over the United Kingdom and Ireland is not significant.

The distribution of the weekly cycle revealed by IASI spatially correlates well with the livestock distribution in Europe^30,31^. The Emissions Database for Global Atmospheric Research (EDGAR) v5.0^39^ consistently reports the largest NH_3_ emissions due to manure management over northwestern Europe, the Po Valley and the Brittany-Pays de la Loire regions (Fig. 2c). The latter accommodate 70 and 60% of the respective pig and poultry population in France^40^. Pig farming is responsible for the largest proportion of manure production in the region^40^. Panels c–d of Fig. 2 also strongly suggest that the reduction in IASI NH_3_ total columns reported on Sunday–Monday is due to a weekly cycle in the emissions captured by the manure management rather than by the agricultural soil emission sector of EDGAR. From this we conclude that fertilization preferentially occurs during week days. This is consistent with Sunday being the traditional rest day in Europe but also with regulations on manure spreading which is prohibited on Sunday (and public holidays) in several regions such as Flanders (Belgium)^41^ and Brittany (France)^42^.

### Ground-based verification

**Figure 3:**
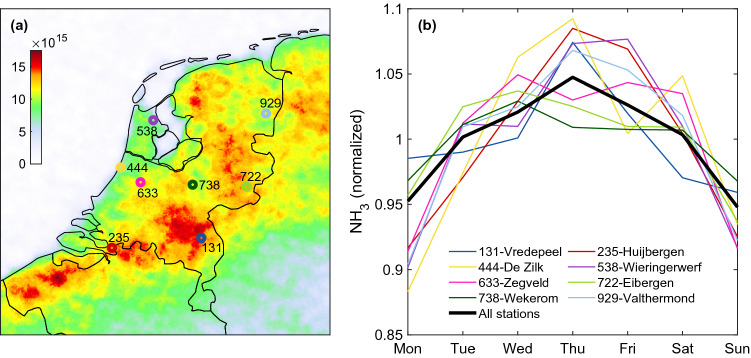
(**a**) Location of the National Air Quality Monitoring Network (LML) stations in the Netherlands superimposed on the IASI-NH_3_ (molec cm$^{-2}$) oversampled distribution (0.01$^{\circ }$ $\times$ 0.01$^{\circ }$ grid, 2008–2020). (**b**) Normalized NH_3_ day of the week time-series measured at the height LML sites and presented individually (coloured lines) and all sites considered together (black line).

To independently confirm the results obtained using the satellite data, we investigate measurements performed from ground. The LML network reports hourly observations of NH_3_ surface concentrations. Figure 3a shows the location of the LML sites used in this study, which are superimposed on the IASI oversampled distribution. The normalized daily variations over the week reveal a marked weekly cycle for each individual site (coloured lines of Fig. 3b) as well as for the network taken as a whole (black line). Consistent with what has been reported from space, the minimal surface concentrations are observed on Sunday–Monday. As far as we are aware of, it is the first time that such a weekly cycle in NH_3_ abundances is reported in the Netherlands. Its amplitude at the national scale is 0.96 μg m$^{-3}$ and the average of Sunday concentrations is 5% below the weekly average. This weekly temporal pattern varies from site to site. Vredepeel (blue, 131) presents by far the largest weekly cycle in absolute terms, with a difference of 2.38 μg m$^{-3}$ between the weekly minimum and maximum. This is not surprising in view of the high emission sources surrounding the measurement site. Likewise, the lowest weekly cycle amplitude (0.47 μg m$^{-3}$) is found in De Zilk (yellow, 444), a coastal site which reports the lowest NH_3_ value in the entire network. However, it is worth noting that in relative terms, it is the site with the largest difference (21%) between its minimum on Monday and its maximum on Thursday.

**Figure 4:**
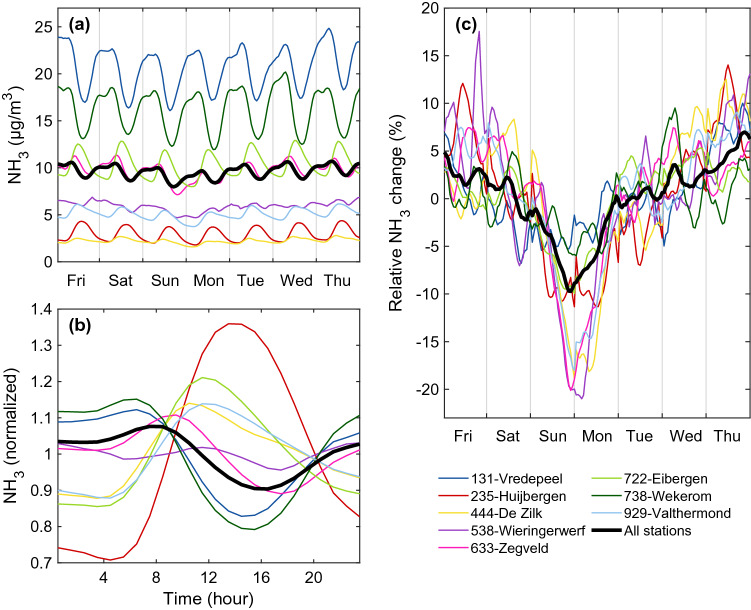
(**a**) NH_3_ hourly surface concentrations (μg m$^{-3}$) for each individual site (coloured lines) from the National Air Quality Monitoring Network (LML) and considering all the sites together (black line). (**b**) Normalized NH_3_ diel cycle. (**c**) Average relative change of NH_3_ (%) obtained by combining panel (**a**) in relative terms with the corresponding averaged diel cycle shown in panel (**b**) removed.

The high temporal sampling of the LML ground-based measurements allow us to investigate in more detail the identified weekend effect. The hourly variability of NH_3_ surface concentrations is shown on Fig. 4a for the entire week, for each station (coloured lines) and across the entire LML network (black line). The smoothness is due to the large amount of LML data considered and the running mean with a window of five hours applied for better visualisation. The diel cycle (Fig. 4b, in relative terms) is markedly different from sites located in source regions, such as Vredepeel (blue, 131) and Wekerom (olive green, 738), and remote sites such as Huijbergen (red, 235) and De Zilk (yellow, 444). In high-emission regions, surface concentrations decrease during daytime, due to increasing wind speed and more favourable conditions for mixing in the planetary boundary layer, and then increase again at the end of the day. By contrast, the background sites are characterized by increasing concentrations during the day, mainly due to transport from source regions^32^.

To highlight the gradual variation of NH_3_ surface concentrations over the course of the week, we remove the average diel cycle from each day to obtain the hourly time-series shown in Fig. 4c in relative terms. While a moderate decrease is initiated from the Friday, the strong decline in NH_3_ occurs on Sunday. Atmospheric concentrations reach a minimum late evening on Sunday and early morning on Monday, with a reduction close to 10%, and are highest on Thursday evening. From all the sites, Zegveld (pink, 633) has one of the largest cycle in both relative and absolute values. Being located in an area dominated by dairy farming with grazing, it is likely the site most influenced by manure application on grassland. The remote sites De Zilk (yellow, 444), Wieringerwerf (purple, 538), Valthermond (light blue, 929) also present a strong weekend effect in relative terms. Vredepeel (blue, 131) and Wekerom (olive green, 738) exhibit generally the same pattern, and are sites influenced by animal housings located close by, which explains the less pronounced decrease during the weekend. The Sunday–Monday minima observed in the ground-based data confirm the timing of the weekend effect reported by IASI, and therefore rejects the hypothesis of the morning overpass time of the satellite being responsible for the late weekend effect observed in the columns.

### Weekly cycle seasonality

**Figure 5:**
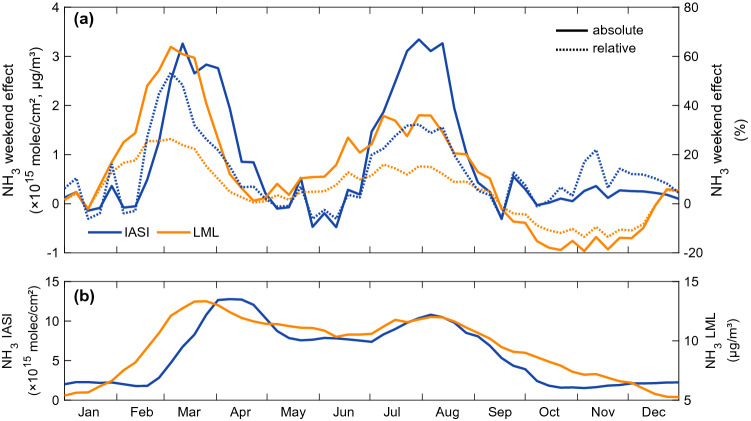
(**a**) Time-series of the absolute (solid lines, molec cm$^{-2}$ and μg m$^{-3}$) and relative (dashed lines,%) weekend effect. The IASI time-series (blue) is based on the morning NH_3_ column satellite observations (2008–2020) over the northwestern European region (50$^{\circ }$N–55$^{\circ }$N; 2$^{\circ }$E–15$^{\circ }$E) and the National Air Quality Monitoring Network (LML) time-series (orange) is based on the NH_3_ surface measurements at the eight stations in the Netherlands. (**b**) Weekly average time-series of the satellite NH_3_ total columns (blue, 10$^{15}$ molec cm$^{-2}$) and surface concentrations (orange, μg m$^{-3}$).

In this section, we investigate the variability of the weekend effect as a function of the time of the year. The bottom panel of Fig. 5 shows the weekly average NH_3_ time-series based on the IASI satellite columns over northwestern Europe (blue) and the ground-based LML surface concentrations in the Netherlands (orange). Both observational datasets show distinct spring and late-summer maxima, corresponding to the two main fertilization periods in Europe^43^. NH_3_ abundances are however high throughout the entire spring–summer period due to agricultural activities and temperature dependent volatilization of NH_3_^44^.

The top panel in Fig. 5 shows the time-series of the weekend effect (with respect to Sunday–Monday) calculated both in absolute (solid lines) and relative (dashed lines) terms. Two distinct peaks are observed, around March–April (weeks 8–16) and a second one around July–August (weeks 27–35). These coincide with the fertilization periods mentioned above, but the seasonality in the weekend effect is much stronger than that seen in the abundances. In spring, a decrease of up to 26% is observed in the LML surface concentrations measured in the Netherlands. A concomitant but even stronger decrease of 53% is seen on Sunday–Monday in the satellite data. The timing of these events strongly supports the conclusion that the weekly effect is largely driven by fertilization.

In most European countries, the time of the year when fertilizers can be applied is in fact tightly regulated^45^. In the Netherlands for instance, application of nitrogen fertilizer is only allowed from February 1 to September 15. Manure application is furthermore regulated in finer detail in the same periods depending on the type of manure (slurry or solid) and the type of land (grassland or arable land)^46^. In Belgium, spreading of nitrogen fertilizers is in general only allowed between February 16 and August 31^41^. The regulation on the timing of application in Germany, is also based on blocking periods, including winter months and depending on fertilizer type and land type^47^. These regulations explain the absence of an observed weekly cycle in the autumn/winter months.

The temporal shift between the NH_3_ abundances and the weekend effect provides also information on the relative contribution of the source processes at play. With a decrease down to 32% in the satellite columns and to 16% in the ground-based surface concentrations in summer, the second peak of the weekend effect is in relative terms substantially lower in both datasets than the first peak. This is explained by the fact that in early spring, fertilization is the dominant source of atmospheric NH_3_. Manure application is also larger in spring than in summer and the spreading period is more condensed^48,49^. By contrast, the summer fertilization peak occurs during a broader period, when other sources also substantially contribute to the ambient NH_3_ abundances.

## Conclusions and perspectives

In previous studies, the temporal variability of atmospheric NH_3_ has been constrained at diurnal and seasonal scale. Likewise, long-term trends have been derived by exploiting over a decade of satellite observations. The remaining gap addressed by the current study is the analysis of the daily variability over the course of the entire week. Here we identified for the first time an unambiguous weekly cycle in the NH_3_ total columns derived from the IASI satellite measurements over Europe. The main weekly temporal pattern consists of decreasing abundances starting on Friday-Saturday, minima observed on Sunday–Monday, and a building up of the abundances during the other week days. The observed weekend effect is most pronounced in northwestern Europe and Brittany, but is also present to a lesser extent in the Po and Ebro Valleys. The weekly cycle revealed from space is confirmed using measured surface concentrations from the Dutch LML ground-based network. While the LML sites are very diverse (from remote to source regions), each of them presents a decrease of NH_3_ on Sunday–Monday.

The intra-annual variability of the weekend effect shows two peaks, corresponding to periods of manure and fertilizer application in Belgium, the Netherlands and Germany. On a yearly basis a weekend effect of 15% is observed in the satellite data over northwestern Europe, increasing to 53% in spring. This reduction on Sunday-Monday is less pronounced on ground, with a maximum drop of 26% during the same season. While NH_3_ emissions from road vehicles have been shown to be underestimated in current inventories^50,51^, the absence of a clear weekly cycle reported by IASI over European cities suggests that traffic currently does not play a dominant role in NH_3_ abundances in the urban environment. Meteorological factors (such as temperature, wind or rain) also affect the presence of NH_3_ in the atmosphere^44^, and one could wonder whether weekly cycles in the former could potentially have an effect on the observed weekly cycle of NH_3_. Small weekly cycles in meteorology have indeed been reported, but mostly over large cities^52^, for which we observed no significant weekend effect in the NH_3_ total columns. Also, outside megacities, we rule out a significant contribution of meteorological parameters, given the very strong correlation of the observed spatial and temporal patterns with that of fertilizer and manure application.

Not shown in this work, we also performed a global analysis with the satellite data. Moderate weekly cycles were identified in some parts of the United States and China, but these were much weaker and less spatially consistent than those found in Europe. The results of this study highlight the importance for Europe of properly taking into account weekly variability of NH_3_ in bottom-up inventories and atmospheric modelling to better represent the NH_3_ atmospheric evolution and related impacts on human and environment health. Future satellite missions, including the IRS geostationary satellite instrument (https://www.eumetsat.int/mtg-infrared-sounder) that will offer hourly measurements, will allow for an even better characterisation of short temporal scale changes in NH_3_ abundances.

## Data Availability

The IASI-NH_3_ datasets are available from the Aeris data infrastructure (http://iasi.aeris-data.fr/NH3). It is also planned to be operationally distributed by EUMETCast under the auspices of the EUMETSAT Atmospheric Monitoring Satellite Application Facility (AC-SAF; http://ac-saf.eumetsat.int).
